# Yap1-2 Isoform Is the Primary Mediator in TGF-β1 Induced EMT in Pancreatic Cancer

**DOI:** 10.3389/fonc.2021.649290

**Published:** 2021-05-19

**Authors:** Chao Gao, Mei-Yu Quan, Qian-Jie Chen, Ruo Yang, Yuanyuan Wu, Jia-Yu Liu, Zhong-Yuan Lin, Xue Li, Jue-Ting Cai, Tian-Fang Jiang, Le Xu, Majid Mossahebi-Mohammadi, Qiang Guo, Jin-San Zhang

**Affiliations:** ^1^ Institute of Life Sciences, Wenzhou University, Wenzhou, China; ^2^ The Key Laboratory of Interventional Pulmonology of Zhejiang Province, The First Affiliated Hospital of Wenzhou Medical University, Wenzhou, China; ^3^ Department of Pharmacy, Cangnan Hospital Affiliated to Wenzhou Medical University, Wenzhou, China; ^4^ International Collaborative Center on Growth Factor Research, and School of Pharmaceutical Sciences, Wenzhou Medical University, Wenzhou, China; ^5^ Eye Hospital, Wenzhou Medical University, Wenzhou, China; ^6^ Division of Respiratory Medicine, Taizhou Enze Hospital, Taizhou, China

**Keywords:** YAP1 isoforms, epithelial-mesenchymal transition, pancreatic cancer, TGF-β, AKT signaling

## Abstract

Pancreatic ductal adenocarcinoma (PDAC) is the most aggressive human malignancy and intrinsically resistant to conventional therapies. YAP1, as a key downstream effector of the Hippo pathway, plays an important role in tumorigenesis including PDAC. Alternative mRNA splicing of YAP1 results in at least 8 protein isoforms, which are divided into two subgroups (YAP1-1 and YAP1-2) based on the presence of either a single or double WW domains. We investigated the functions and regulatory mechanisms of YAP1-1 and YAP1-2 in PDAC cells induced by TGF-β to undergo epithelial-to-mesenchymal transition (EMT). CRISPR-Cas9 and shRNA were used to silence YAP1 expression in pancreatic cancer cells. Re-constituted lentivirus mediated overexpression of each single YAP1 isoform was generated in the parental knockout L3.6 cells. EMT was induced by treatment with TGF-β, EGF and bFGF in parental and the constructed stable cell lines. Western blot and qPCR were used to detect the expression of EMT markers. Scratch wound healing and transwell assays were used to detect cell migration. The stability and subcellular localization of YAP1 proteins were determined by Western blot analysis, immunofluorescence, as well as ubiquitination assays. We showed that TGF-β, EGF and bFGF all significantly promoted EMT in PDAC cells, which was inhibited by knockdown of YAP1 expression. Interestingly, YAP1-1 stable cells exhibited a stronger migratory ability than YAP1-2 cells under normal culture condition. However, upon TGF-β treatment, L3.6-YAP1-2 cells exhibited a stronger migratory ability than L3.6-YAP1-1 cells. Mechanistically, TGF-β treatment preferentially stabilizes YAP1-2 and enhances its nuclear localization. Furthermore, TGF-β-induced EMT and YAP1-2 activity were both blocked by inhibition of AKT signaling. Our results showed that both YAP1-1 and YAP1-2 isoforms are important mediators in the EMT process of pancreatic cancer. However, YAP1-2 is more important in mediating TGF-β-induced EMT, which requires AKT signaling.

## Introduction

Pancreatic ductal adenocarcinoma (PDAC), is highly fatal due to its aggressive biology nature and intrinsic resistance to conventional therapies (1). Yes-associated protein 1 (YAP1), together with WW domain-containing transcription regulator protein 1 (WWT1, also called TAZ), function as the main downstream effectors of the Hippo pathway. YAP1 plays critical roles in tissue homeostasis and regulation of organ size. The Hippo signaling is also a tumor suppressor pathway, while YAP1 has been identified as an oncogene in various malignancies associated with tumor progression and poor prognosis (2). YAP1 overexpression is detected in the early stages of pancreatic carcinogenesis (3). Interestingly, YAP1 has been shown to be involved in both oncogenic KRAS-dependent and KRAS-independent cancer-promoting activities (4, 5). In recent years, YAP1 has been reported to participate in Epithelial-to-mesenchymal transition (EMT) process of tumor cells (6, 7).

Through alternative mRNA splicing, the human *YAP1* gene generates at least eight isoforms that differ in the regions of the 2^nd^ WW domain and the transcriptional activation domain (TAD) (8). These isoforms can be divided into YAP1-1, which contains one WW domain, and YAP1-2, which contains two WW domains (9). The WW domain consists of incomplete duplicates of 30-40 amino acid residues, of which two invariant tryptophan residues mediate specific interactions with partners with short proline-rich sequences (9, 10). The WW domain of YAP1 is involved in complex formation with PPxY motif-containing proteins (where P is proline, x is any amino acid and Y is tyrosine) (11), such as LATS1/2 (12), AMOT (13), PTCH1 (14), ZEB1 (15), etc. Previous studies, including our own, revealed that the presence of single or double WW domains could influence the interactions of YAP1 with these proteins such as LATS (16). Although the mRNA sequences encoding different isoforms have been reported, the biological and functional differences of various protein isoforms of YAP1 have just begun to be appreciated.

EMT is a cellular reprograming process during which cells lose their epithelial traits and gradually acquire mesenchymal characteristics (17), such as downregulation of E-Cadherin and upregulation of Vimentin, resulting in weakened adhesion and enhanced motility (18). During EMT, remodeling of cell-cell and cell-extracellular matrix interactions leads to the detachment of epithelial cells from their original sites. This is crucial to the early-stage dissemination of cancer cells and is pivotal for the invasion and metastasis. Moreover, EMT has been reported to confer increased chemoresistance in cancer cells, including PDAC (19, 20). Transforming growth factor-beta (TGF-β) (21), epidermal growth factor (EGF) (22), and fibroblast growth factor (FGF) (23), are all well-known cytokines that promote the EMT phenotype (24). TGF-β activates Smad2 and Smad3, which subsequently bind to Smad4 and then translocate to the nucleus to regulate gene expression (25). TGF-β can also activate the PI3K/AKT pathway to promote EMT (26). YAP1 plays a multitude of roles as the key downstream effector of Hippo signaling. It promotes tumor development and progression, as well as EMT and drug resistance in PDAC (27).

In this study, we investigated whether YAP1 contributes to TGF-β-induced EMT in PDAC cells and the potential difference between the YAP1 isoforms that mediate such activities. Our results indicated that, with TGF-β treatment, YAP1-2 exhibited stronger effects than YAP1-1 in promoting EMT in PDAC cells. Mechanistically, we showed that TGF-β treatment activates the AKT pathway to preferentially stabilize YAP1-2 and promote its nuclear localization.

## Materials and Methods

### Cell Culture and Treatment

Human pancreatic cancer cell lines L3.6, PANC1, PATU89.88T, and rat pheochromocytoma cell line PC12 were obtained from ATCC. The human embryonic kidney cell line HEK293T was obtained from the Shanghai Cell Bank of the Chinese Academy of Sciences. All cells were cultured in RPMI 1640 (Gibco) or DMEM (Gibco) medium with 10% fetal bovine serum (FBS, Gibco) at 37°C in standard conditions (5% CO_2_, 95% air).

### Transwell Assays

A total of 1×10^5^ L3.6 cells were seeded in the upper chamber of a transwell membrane (8 μm pore size) in 24-well plates. FBS medium (10%) was added to the lower chamber as an attractant, while 1% FBS medium was added to the upper chamber. After 24 h of incubation, the cells were fixed with paraformaldehyde (PFA) and stained with crystal violet dye. The number of stained cells was counted under a phase-contrast microscope (Leica, Germany).

### Western Blot

Total protein was extracted from PDAC cells for Western blot analysis. The cells were lysed with lysis buffer (Beyotime, P0013) containing phosphatase inhibitors and phenylmethanesulfonyl fluoride (PMSF, Beyotime, ST506). The samples were fractionated by SDS-PAGE gels and transferred to microporous polyvinylidene difluoride (PVDF) membranes (Roche, 3010040001). The membranes were blocked with 5% skim milk for 1 h and incubated with primary antibodies overnight at 4°C. Antibodies against GAPDH (10494-1-AP) and anti-β-actin (20536-1-AP) were purchased from Proteintech. Other primary antibodies including anti-YAP (CST, 14074), anti-E-Cadherin (CST, 3195), anti-Vimentin (CST, 5741), anti-phospho-Smad2 (CST, 8828), anti-Smad2/3 (CST, 8685), anti-phospho AKT (CST, 4060), and anti-AKT (CST, 9272) were the products of Cell Signaling Technology. The membranes were then probed with secondary antibodies: goat anti-mouse IgG (H+L)-HRP or goat anti-rabbit IgG (H+L)-HRP (Bioworld).

### Scratch Healing Assay

The cells were seeded in 6-well plates, and 10 μl pipette tips were used to make a scratch to assess regeneration and repair of the cells. The wound images were taken at 0 h, 24 h, 48 h, and 72 h, respectively.

### Immunofluorescence

The cells were seeded in 24-well plates at the appropriate density for 12 h. They were fixed with 4% PFA for 15 min and permeabilized with 0.1% Triton X-100 for 7 min. They were then blocked with 5% normal sheep serum before incubating with primary antibody (1:100) for 2 h at room temperature followed by incubation with the secondary antibodies for 1 h at room temperature (Goat anti-Mouse IgG H&L, Abcam, 1:1000). After washes with PBS, the cells were mounted with Gold Antifade with DAPI (Invitrogen, P36931).

### RNA Isolation, Real-Time PCR and YAP1 Isoform Detection

Total RNA was extracted with RNAiso Plus (TaKaRa, JPN). The PrimeScript RT Reagent Kit (TaKaRa, JPN) was used for cDNA synthesis. Real-time PCR was carried out with the CFX96 Real-Time System (Bio-Rad) and SYBR Premix Ex Taq (TaKaRa, JPN). The gene-specific primers used in this study were as follows: Vimentin (forward: 5’-GAGGATCTGGAATTCGGATCC-3’, reverse:5’- ACGCGTCGACTTATTCAAGGT-3’); E-Cadherin (forward: 5’-AATGCCGCCATCGCTTAC-3’, reverse: 5’-ACCAGGGTATACGTAGGGAAACTCT-3’); and GAPDH (forward: 5’-ACATCGCTCAGACACCATG-3’, reverse: 5’-TGTAGTTGAGGTCAATGAAGGG-3’). All values were normalized to GAPDH.

### Lentiviral Packaging, Transduction, and Selection of Stable Cells

Lentiviral packaging, host cell infection and pLKO-shRNA stable PDAC cell selection with puromycin were performed as previously described (28). For the stable reconstituted expression of YAP1-specific isoforms, lentiviral particles carrying pLenti6.3-Flag-YAP1 cDNA encoding YAP1-1γ or YAP1-2γ were used to infect L3.6-KoYAP1 cells. The cells were then selected in culture medium supplemented with blasticidin (5 μg/ml), and the pooled blasticidin-resistant cells were used as stable overexpression cells.

### Ubiquitination Assay

HEK293T cells were transfected with the *Flag-YAP1* and *HA-UbI* vectors and incubated with 20 μM MG132 for 4 h before harvesting. The cells were washed twice with prechilled PBS and lysed in 120 μL of lysis buffer (10% glycerol, 1% SDS, 62.5 mM Tris-HCl (pH 6.8), 1 mM iodoacetamide and 10 mM NEM). Cell lysates were boiled for 15 min and then diluted with NTEN lysis buffer freshly supplemented with protease and deubiquitination inhibitors at a 1:9 ratio. The cell lysates were immunoprecipitated with anti-Flag M2 agarose beads (Sigma, USA), and the immune complexes were subjected to Western blotting.

### Statistical Analysis

Statistical analyses were performed using GraphPad Prism 8 (GraphPad Software, San Diego, CA, USA) and SPSS 19.0 software (SPSS, Inc., Chicago, USA). p values showing differences were calculated by an unpaired two-tailed t-test, and those showing no differences were calculated by a one-tailed t-test.

## Results

### YAP1 Contributes to EMT Phenotype in PDAC Cells

**Figure 1:**
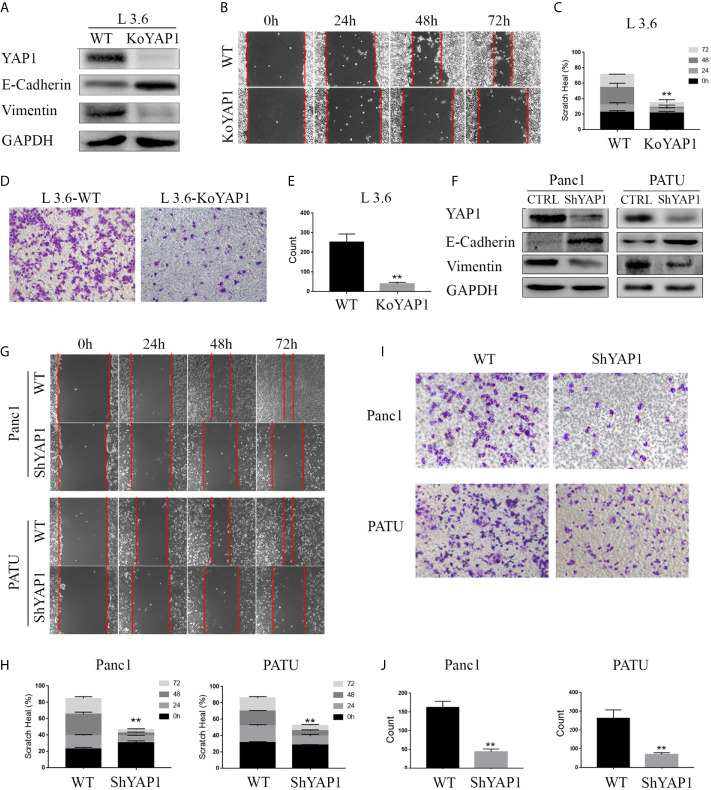
YAP1 contributes to EMT phenotype in PDAC cells. **(A)** Western blot analysis of YAP1 and EMT marker expression in L3.6 wild type (WT) and YAP1 knockout cells (KoYAP1). **(B–E)** The migration and invasion ability of L3.6-WT and L3.6-KoYAP1 were detected by scratch healing assay **(B)** and transwell assay **(D)**. Statistical analysis of the scratch healing assay **(C)** and transwell assay **(E)**. **p < 0.001. **(F–J)** Different PADC cell lines were used to verify the effect of YAP1. EMT markers (E-Cadherin and Vimentin) and YAP1 knockdown efficiency were determined by western blot **(F)**, migration and invasion of the indicated cell lines were detected by scratch healing assay **(G)** and transwell assay(I). Statistical analysis of the scratch healing assay **(H)** and transwell assay **(J)**. **p < 0.001.

We determined the role of YAP1 in the EMT of pancreatic cancer cells by examining the expression of EMT markers in a YAP1 knockout cell line previously generated in L3.6 (16). The Western blot results showed that deletion of YAP1 led to increased Vimentin, but decreased E-Cadherin expression (**Figure 1A**). Furthermore, wound healing and transwell assays indicated that the migratory and invasive potential were diminished in YAP1-KO cells (**Figures 1B–E**). We further verified that YAP1 knockdown was associated with reduced migration and invasion capabilities in PANC1 and PATU8988T cell lines. YAP1 knockdown led to increased Vimentin, but decreased E-Cadherin expression in PANC1 and PATU8988T (**Figure 1F**). Suppression of YAP1 expression in these cells were achieved by lenti shRNA, which also led to decreased migration and invasion based on wounding and transwell assays (**Figures 1G–J**). Therefore, YAP1 expression correlated with migration and invasion in three independent pancreatic cancer cell lines.

### YAP1 Mediates Growth Factor-Induced EMT in L3.6 Cells

**Figure 2:**
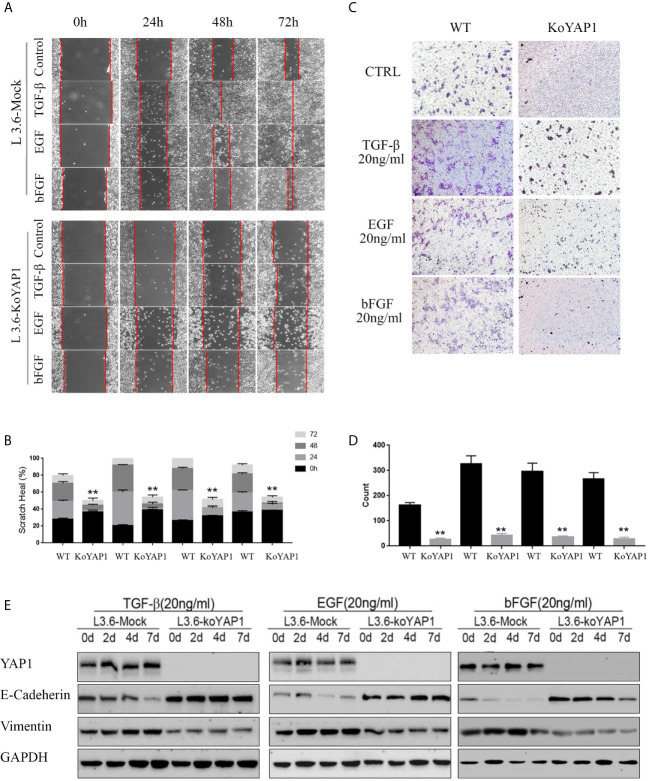
Knockout of YAP1 blocks EMT phenotype induced by TGF-β, bFGF and EGF. L3.6-WT and L3.6-KoYAP1 cells were treated with TGF-β (20ng/ml), EGF (20ng/ml), and bFGF (20ng/ml), respectively, for 72 h. Cell migration and invasion were detected by scratch healing assay **(A)** and transwell assay **(C)** and the expression of EMT markers by western blot **(E)**. Statistical analysis of the scratch healing assay **(B)** and transwell assay **(D)**. **p < 0.001.

Given that YAP1 knockout or knockdown inhibited the invasion and migration of PDAC cells, we hypothesized that YAP1 may also play a role in growth factor-induced EMT. As shown in **Figures 2A, B**, treatment with TGF-β (20ng/ml), EGF (20ng/ml), and bFGF (20ng/ml) significantly promoted healing of parental L3.6 cells in the wounding assay, which were significantly inhibited in L3.6-KoYAP1 cells. Transwell assays confirmed these results (**Figures 2C, D**). Western blot analyses were used to detect the EMT-associated proteins E-Cadherin and Vimentin, and the results indicated that YAP1-KO effectively prevented the occurrence of EMT characteristics induced by these growth factors (**Figure 2E**). In summary, the results showed that YAP1 expression plays an important role in the EMT induced by different growth factors.

### Identification of YAP1-2 as the Major Isoform in TGF-β-Induced EMT

**Figure 3:**
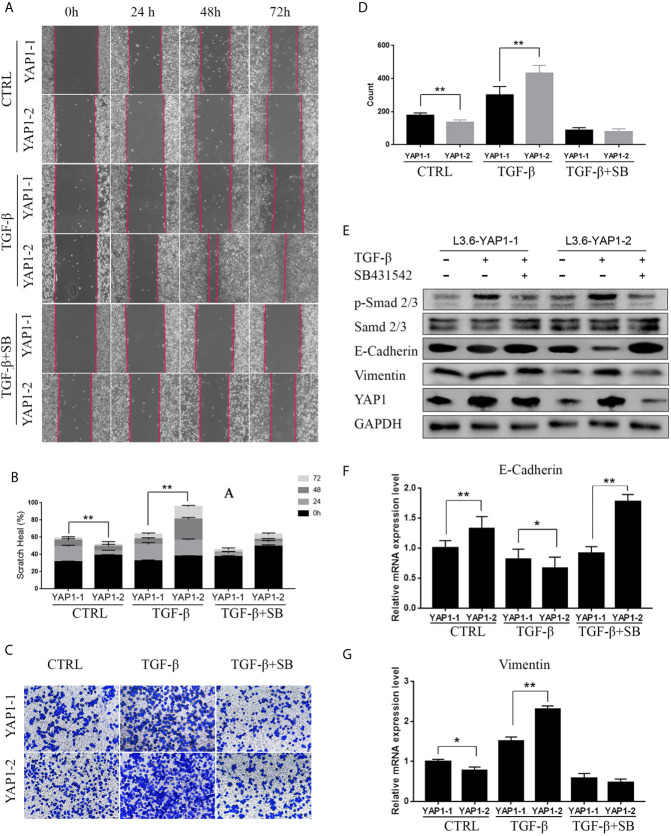
YAP1-2 is the major functional isoform in mediating TGFβ-induced EMT. **(A, C)** Cells were pretreated with 20ng/ml TGF-β and 2.5μM SB431542 for 72 h, the scratch healing assay **(A)** and transwell assay **(C)** were performed to detect cell migration in L3.6-YAP1-1 and L3.6-YAP1-2 stable lines. **(B, D)** Statistical analysis of the scratch healing assay **(B)**, and transwell assay **(D)**. **p < 0.001. **(E–G)** Cells were treated with 20ng/ml TGF-β and 2.5μM SB431542 for 72 h, Western Blot **(E)** and qPCR **(F, G)** were performed to detect the expression of EMT related markers in L3.6-YAP1-1 and L3.6-YAP1-2 cells. SB431542 is abbreviated as “SB”. *p < 0.05, **p < 0.001.

The above results suggest YAP1 as an important mediator in these growth factor-induced EMT phenotype. We next wanted to determine if the YAP1 protein isoforms presented distinct or redundant roles in EMT process with a focus on TGF-β. Scratch assays were performed with L3.6-YAP1-1 and L3.6-YAP1-2 stable cells. These cells were generated in our previous study on YAP1 knockout L3.6 cells with reconstituted overexpression of single YAP1 isoforms (16). The results revealed that YAP1-1 has a stronger stimulatory effect on EMT than YAP1-2 in none treated cells. However, upon induction by TGF-β, L3.6-YAP1-2 cells exhibited stronger migration than L3.6-YAP1-1 cells (**Figures 3A, B**). SB431542, a TGF-β receptor inhibitor, largely abolished TGF-β-induced EMT in both L3.6-YAP1-1 and L3.6-YAP1-2 cells. Consistent results were obtained in the transwell assays (**Figures 3C, D**). Upon examining the TGF-β signaling and EMT markers, we found YAP1-2 cells possessed higher E-Cadherin expression when untreated than YAP1-1. Interestingly, the E-Cadherin expression in YAP1-2 cells was significantly inhibited by TGF-β treatment, but that of YAP1-1 was minimally impacted. The trend of Vimentin was the opposite, and all changes were reversed by SB431542 (**Figures 3E–G**). Also, we showed that blocking YAP1 using Verteporfin, a YAP1 inhibitor, led to a significant change in the expression of E-Cadherin and Vimentin in L3.6-YAP1-2 cells (**Figure S2**). In short, the results proved that the YAP1-2 is more potent than YAP1-1 in mediating TGFβ-induced EMT in pancreatic cancer cells.

### TGF-β Mainly Promotes YAP1-2 Stability and Its Nuclear Localization

**Figure 4:**
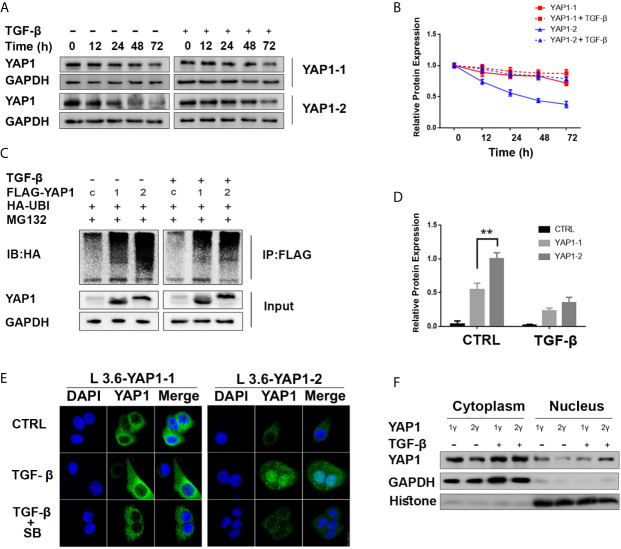
TGF-β promotes YAP1-2 stability and nuclear localization. **(A)** L3.6-YAP1-1 and L3.6-YAP1-2 cells were cultured under the low-density condition for 3 days to accumulate YAP1 proteins with or without TGF-β (20ng/ml) treatment. The cells were then transferred to 3.5 cm dishes in high-density conditions to trigger YAP1 degradation. Whole-cell lysates from L3.6-YAP1-1 and L3.6-YAP1-2 cells were collected at indicated time points and subjected to Western blotting to detect the abundance of YAP1. **(B)** Statistical analysis of **(A)**. **(C)** HA-tagged ubiquitin was co-transfected with either Flag-YAP1-1 or YAP1-2 into HEK293T cells as indicated with Flag-YFP as a control. The transfected cells were treated with 20ng/ml TGF-β for 24 h. YAP1 ubiquitination was determined by immunoprecipitation (IP) for Flag and immunoblotting for HA. **(D)** Statistical analysis of **(C)**. **p< 0.001. **(E)** Cells were pretreated with 20ng/ml TGF-β and 2.5μM SB431542 for 72 h, immunofluorescence was performed to reveal the expression and subcellular localization of L3.6-YAP1-1 and L3.6-YAP1-2 cells. **(F)** Cells were pretreated with 20ng/ml TGF-β for 72 h, extraction of cytoplasmic and nuclear proteins was then performed to detect the distribution of YAP1 in L3.6-YAP1-1 and L3.6-YAP1-2 by immunoblot analysis.

YAP1 stability and nuclear translocation represent a key regulatory mechanism downstream of Hippo signaling. Western blot analysis revealed that the YAP1-1 protein was more stable than the YAP1-2 protein under normal conditions, but the stability of the YAP1-2 protein was preferentially improved after TGF-β treatment (**Figures 4A, B**). Immunoprecipitation assay results indicated that the ubiquitination level of YAP1-2 was significantly higher than that of YAP1-1. Although TGF-β treatment significantly decreased the ubiquitination levels of both isoforms, the gap between YAP1-1 and YAP1-2 was narrowed down significantly (**Figures 4C, D**), and TGF-β treatment also stabilized both YAP1-1 and YAP1-2 proteins induced by verteporfin (**Figure S2**). These results suggest that the stability of both proteins was upregulated after induction, but YAP1-2 changed more significantly than YAP1-1. Subsequently, we examined the subcellular localization of the different isoforms of YAP1 with immunofluorescence. Increased nuclear translocation of YAP1-2 was observed after TGF-β treatment, and the process could be inhibited by SB431542, whereas the localization of YAP1-1 only changed slightly (**Figure 4E**). Western blot analysis confirmed that YAP1-2 protein expression was upregulated in both the nucleus and cytoplasm after induction, but was more prominent in the nucleus (**Figure 4F**). In summary, the data demonstrated that both the stability and location of YAP1-2 changed significantly after TGF-β induction.

### AKT Inhibition Abolishes the Function of YAP1-2 in TGF-β-Induced EMT

**Figure 5:**
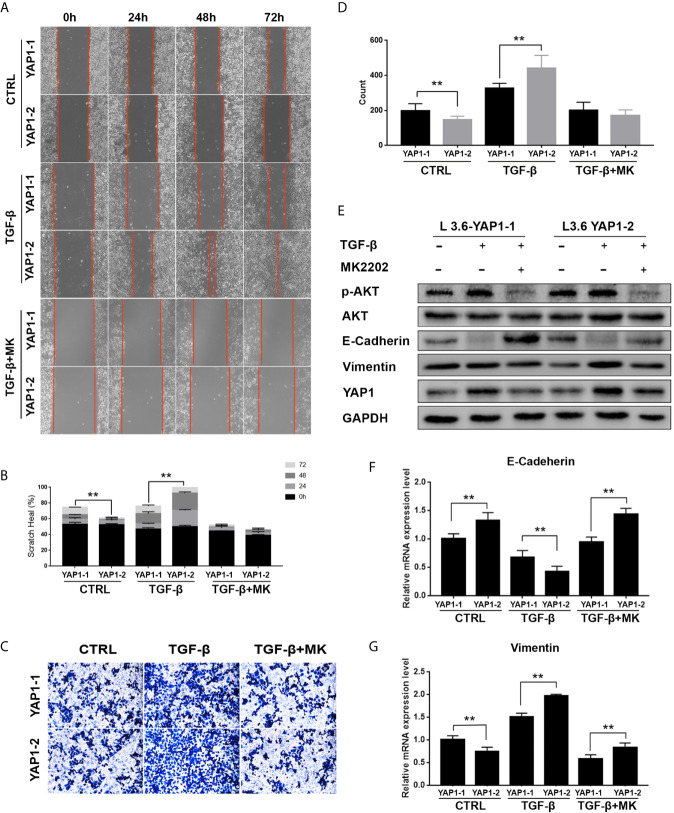
Inhibition of AKT signaling abolishes the activity of YAP1-2 in TGF-β induced EMT. **(A, C)** L3.6-YAP1-1 and L3.6-YAP1-2 stable cells were pretreated with 20ng/ml TGF-β and 2.5μM MK2206 for 72 h, the scratch healing assay **(A)** and transwell assay **(C)** were performed to determine the migration ability of L3.6-YAP1-1 and L3.6-YAP1-2. **(B, D)** Statistical analysis of the scratch healing assay **(B)** and transwell assay **(D)**. **p < 0.001. **(E, F)** Cells were pretreated with 20ng/ml TGF-β for 72 h, and then with MK2206 (2.5μM) for another 24 h. Western Blot **(E)** and qPCR **(F, G)** were performed to detect the expression of EMT markers and AKT phosphorylation. MK2206 is abbreviated as “MK”. **p < 0.001.

In previous studies, it was reported that YAP1 activity closely correlates with the AKT signaling activation in fibrotic disease (29), wound healing (30), etc. Therefore, we evaluated the role of the AKT pathway in YAP1-related EMT by co-treatment of the cells with MK2206, an AKT phosphorylation inhibitor. As expected, Western blot analysis showed that the phosphorylation of AKT was significantly inhibited by MK2206 treatment (**Figures 5E**, **S1**). Scratch healing assay (**Figures 5A, B**) and transwell assay (**Figures 5C, D**) results indicated that MK2206 treatment significantly inhibited migration and invasion of both L3.6-YAP1-1 and L3.6-YAP1-2 cells with TGF-β-induced EMT. The Western blot and qRT-PCR results showed that MK2206 upregulated E-Cadherin and downregulated Vimentin at both the protein and mRNA levels in the L3.6-YAP1-1 and L3.6-YAP1-2 cells with TGF-β-induced EMT (**Figures 5E–G**, **S1**). Additionally, MK2206 treatment also decreased the protein levels of YAP1-1 and YAP1-2 (**Figures 5E**, **S1**). In summary, YAP1-2 is more important than YAP1-1 in mediating TGF-β-induced invasion and migration of L3.6 cells, a process relies on activation of the AKT pathway.

### AKT Signaling Is Required for TGF-β-Induced YAP1 Stabilization and Nuclear Localization

**Figure 6:**
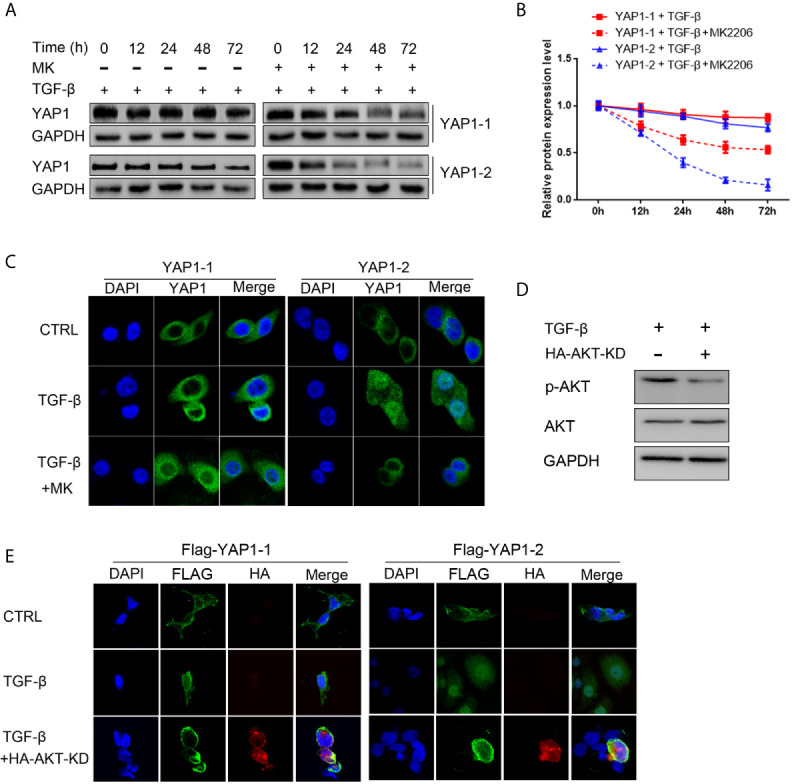
AKT signaling is required for TGF-β induced YAP1 stabilization and nucleus localization. **(A)** L3.6-YAP1-1 and L3.6-YAP1-2 cells were cultured at low-density for 3 days to accumulate YAP1 proteins with or without 20ng/ml TGF-β and 2.5μM MK2206 treatment. The cells were then transferred to 3.5 cm dishes at high-density to trigger degradation. Whole cell lysates were collected at indicated time points and subjected to Western blotting to detect the abundance of YAP1. **(B)** Statistical analysis of **(A)**. **(C)** Cells were treated with 20ng/ml TGF-β and MK2206 for 72 h, immunofluorescence was performed to detect the expression and subcellular localization of YAP1 in L3.6-YAP1-1 and L3.6-YAP1-2 stable cells. **(D)** The HA-AKT(KD) mutant effectively inhibited activation of endogenous AKT. **(E)** HA- AKT(KD) was transfected to PC12 cells, and simultaneously treated with 20ng/ml TGF-β. Immunofluorescence was performed to detect the Tag (Flag) of exogenous YAP1. MK2206 is abbreviated as “MK”.

We determined the potential differential impact of AKT signaling on YAP1 isoform stability and subcellular location in pancreatic cancer cells by examining their expression in response to TGF-β treatment, both with and without the AKT inhibitor. To this end, we chose previously generated L3.6-YAP1-1 and L3.6-YAP1-2 reconstituted expression stable cell lines (16). The cells were seeded at very low density (10^6^ cells/10-cm dish) for 3 days and then trypsinized and replanted at high cell density (2x10^6^ cells/3.5-cm dish) for TGF-β treatment with and without the AKT inhibitor. The results indicated that the stabilizing effect of TGF-β on both YAP1-1 and YAP1-2 were diminished, but much more prominent for YAP1-2 (**Figures 6A, B**). Similarly, inhibition of AKT phosphorylation largely blocked the nuclear localization of YAP1-2 (**Figure 6C**). Therefore, the activity of the AKT pathway is of critical importance for TGF-β promoted EMT, which is mainly mediated by YAP1-2. To further confirm the role of AKT in TGF- β induced EMT, the AKT- Kinase Dead (KD) mutant plasmid was co-transfected with YAP1-1 and YAP1-2, respectively, to PC12 cells. Analysis of fractionated proteins showed that nuclear-translocation of YAP1-2 was inhibited upon AKT-KD co-expression (**Figures 6D, E**).

## Discussion

YAP1 protein isoforms differ within the TAD and WW motifs, two key regions mediating their transcriptional activation and interaction with PPxY motif proteins (8). We recently reported the dichotomy between the mRNA and protein expression, as well as the distinct mechanism of regulation between the YAP1-1 and YAP1-2 isoforms in response to cell density. We proposed that YAP1-1 is more potent in promoting cancer cell malignancy in culture and primary tumor growth *in vivo*, whereas YAP1-2 has a more significant role in promoting metastasis due to its stabilization under low cell contact/density such as in the form of circulating tumor cells (16).

Consistent with our previous findings, the current study further demonstrated that the stability and nuclear localization of YAP1-1 proteins were higher than those of YAP1-2 under quiescent conditions, and correlates with stronger invasion and metastasis. Interestingly, YAP1-2 exhibited stronger promotion of the invasion and metastasis of PDAC cells than YAP1-1 in the process of TGF-β induced EMT, which was associated with a more robust increase in protein stability as well as nuclear localization of YAP1-2 than YAP1-1. Therefore, stabilization of YAP1 proteins, especially the YAP1-2 isoform, contributes to TGF-β induced EMT. However, we cannot rule out the possibility that other mechanisms may also be involved. We have previously demonstrated that the presence of the 2^nd^ WW motif enhances the interactions of both YAP1-1 and YAP1-2 proteins with some PPXY proteins such as LATS1. Importantly, only YAP1-2 is capable of forming *de novo* complex with AMOT and PTPN14. Of note, the WW domain not only mediates binding to negative regulators mainly residing in the cytosol as mentioned above, but it also interacts with nuclear factors such as RUNX (31), ZEB1 (15), P73 (32, 33) and SMADs (34, 35) to alter the targets of YAP1. ZEB1 and SMADs are both important EMT regulators. During EMT, ZEB1 and SMADs are often increased, showing stronger nuclear localization and higher transcriptional activity, a process that we speculate may involve YAP1-2. It is also possible that, in response to TGF-β stimulation, activated YAP1-2 preferentially binds to EMT-related nuclear factors to facilitate their nuclear transport and transcriptional activities, which should be an interesting area for future investigation.

Our data highlight a critical role of activated AKT signaling for the stability of YAP1 in the context of TGF-β stimulation. AKT has been shown to bind directly to YAP1 and enhance its stability. However, this molecule is not the only factor that regulates the stability of YAP1. The main regulation of YAP1 is from the upstream Hippo pathway, which is subjected to regulation by diverse stimuli such as cell-cell contact, mechanical cues, as well as EMT. When cells undergo EMT, they shrink, and cell contact becomes weaker. Under such circumstances, Hippo pathway is suppressed leading to YAP1 stabilization. Given that inhibition of the AKT pathway is known to strongly inhibit EMT phenotype, thus TGF-β stimulated AKT activation may contribute directly and indirectly to the stabilization and the nuclear localization of YAP1.

EMT is a crucial initiating step of tumor metastasis and correlates to the advanced stages of tumor progression. Our data suggest the existence of redundant and unique regulatory mechanisms underlying the functions of YAP1-1 and YAP1-2 in cancer development and progression. We propose that in the early stages, when cancer cells have a low level of malignancy, YAP1-1 is predominant due to its weaker binding with negative regulatory factors than YAP1-2 and mainly promotes cell proliferation. However, when the tumors progress to a higher degree of malignancy after undergoing EMT, YAP1-2 becomes preferentially stabilized, leading to increased nuclear localization and interactions with additional nuclear factors to promote tumor invasion and metastasis. The potential differential interactions of YAP1-2 from YAP1-1 with EMT-promoting nuclear factors, such as SMADs and ZEB1, should be an interesting line of research in the future.

Hippo signaling has emerged as an attractive target for cancer therapy including PDAC (27). YAP1 acts as a central node in relaying Hippo signaling to transcriptional regulation and cancer promotion. Our findings on the unique function and associated regulatory mechanisms between different YAP1 isoforms should facilitate more efficient and precise therapeutic targeting of this critical pathway for cancer therapy.

## Data Availability Statement

The raw data supporting the conclusions of this article will be made available by the authors, without undue reservation.

## Author Contributions

J-SZ and QG conceived the study. M-YQ, RY, CG, J-TC, Q-JC, Z-YL, XL, YW, J-YL, T-FJ, and LX: methodology, data acquisition, and analysis. CG and QG drafted the manuscript. MM-M and J-SZ edited and revised the manuscript. All authors have read and agreed to this version of the manuscript. All authors contributed to the article and approved the submitted version.

## Funding

QG was funded by the National Natural Science Foundation of China (#81702912). J-SZ was partially supported by a start-up package from Wenzhou University.

## Conflict of Interest

The authors declare that the research was conducted in the absence of any commercial or financial relationships that could be construed as a potential conflict of interest.

## Supplementary Material

Supplementary Figure 1 MK2206 treatment of L3.6-YAP1-1 and L3.6-YAP1-2. MK2206 was added to L3.6-YAP1-1 and L3.6-YAP1-2 stable cells. The results showed that the EMT marker did not change after MK2206 treatment.

Supplementary Figure 2 YAP1 inhibition affects the expression of EMT markers. L3.6-YAP1-1 and L3.6-YAP1-2 stable cell lines were treated with Verteporfin, YAP1 specific inhibitor, alone or in combination with TGF-β.
