# Supplementary figures and images for: Yap1-2 Isoform Is the Primary Mediator in TGF-β1 Induced EMT in Pancreatic Cancer
Source: Front Oncol. 2021 May 19;11:649290. doi: 10.3389/fonc.2021.649290 (PMC8170464; doi:10.3389/fonc.2021.649290)

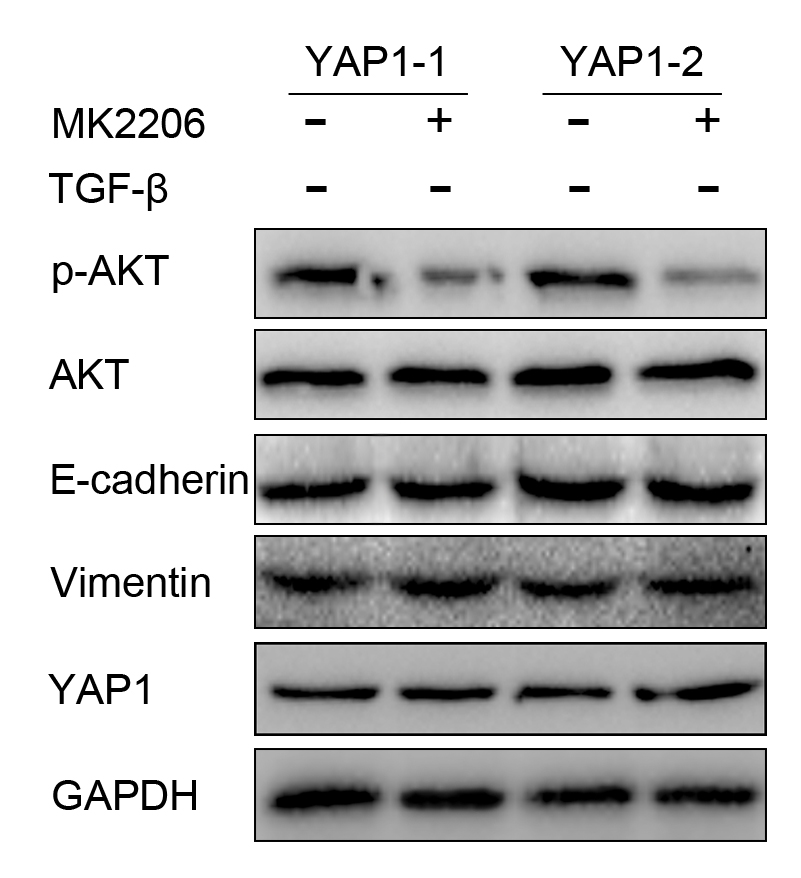

Supplement: Supplementary Figure 1 — MK2206 treatment of L3.6-YAP1-1 and L3.6-YAP1-2. MK2206 was added to L3.6-YAP1-1 and L3.6-YAP1-2 stable cells. The results showed that the EMT marker did not change after MK2206 treatment. [file Image_1.jpeg]

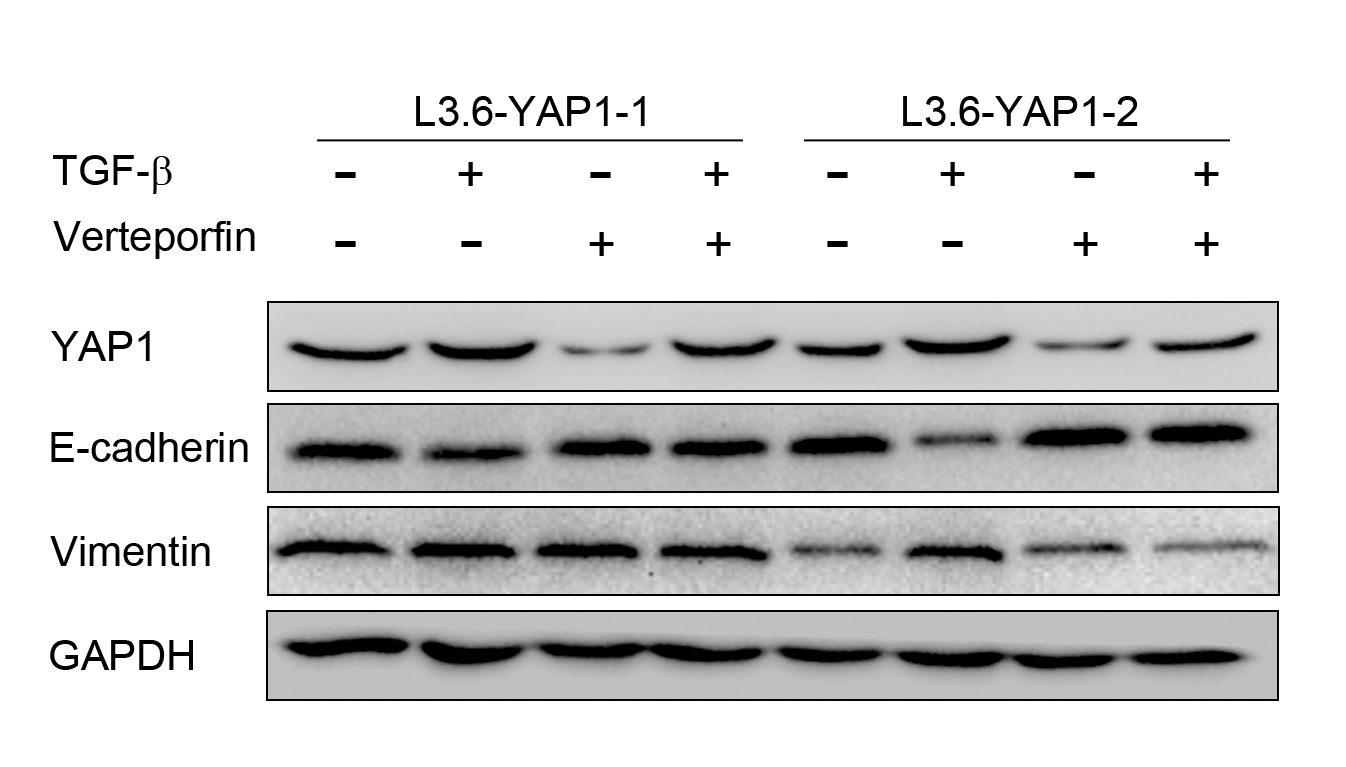

Supplement: Supplementary Figure 2 — YAP1 inhibition affects the expression of EMT markers. L3.6-YAP1-1 and L3.6-YAP1-2 stable cell lines were treated with Verteporfin, YAP1 specific inhibitor, alone or in combination with TGF-β. [file Image_2.jpeg]
